# Supplementary material for: Replication gap suppression depends on the double-strand DNA binding activity of BRCA2
Source: Nat Commun. 2023 Jan 27;14:446. doi: 10.1038/s41467-023-36149-0 (PMC9883520; doi:10.1038/s41467-023-36149-0)
Supplement: Supplementary file 1 — Supplementary Information [file 41467_2023_36149_MOESM1_ESM.pdf]

Replication gap suppression depends on the double-strand DNA binding activity of BRCA2

Supplementary Information: Supplementary Fig. 1-7 and legends and Supplementary Table 1

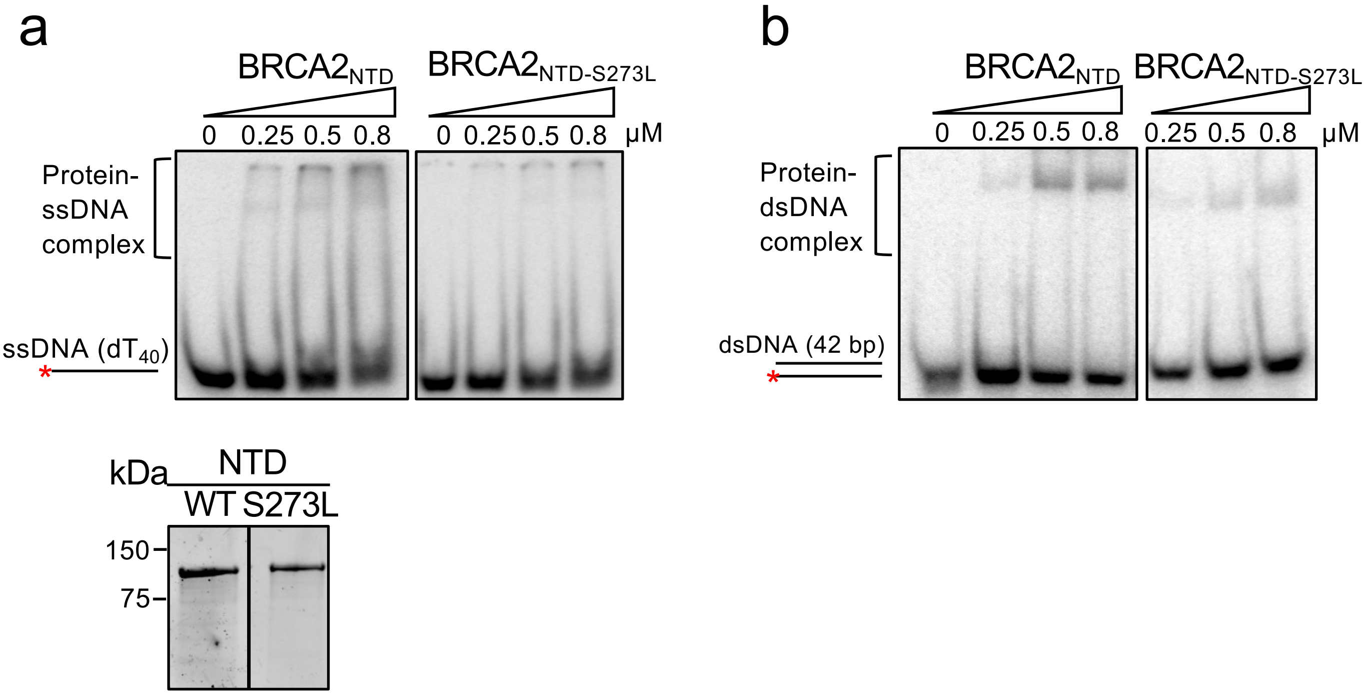

a
BRCA2NTD
BRCA2NTD-S273L
0 0.25 0.5 0.8
0 0.25 0.5 0.8 μM
Protein-ssDNA complex
ssDNA (dT40)
kDa
NTD
WT S273L
150
75
b
BRCA2NTD
BRCA2NTD-S273L
0 0.25 0.5 0.8
0.25 0.5 0.8 μM
Protein-dsDNA complex
dsDNA (42 bp)

Supplementary Figure 1. Relative to Figure 1a. Representative EMSA comparing the binding of $BRCA2_{NTD}$ and $BRCA2_{NTD\text{-}S273L}$ to ssDNA (dT_{40}) (**A**) or dsDNA (42mer) (**B**). Bottom SDS-PAGE gel showing the purified $BRCA2_{NTD}$ and $BRCA2_{NTD\text{-}S273L}$ used for the EMSAs.

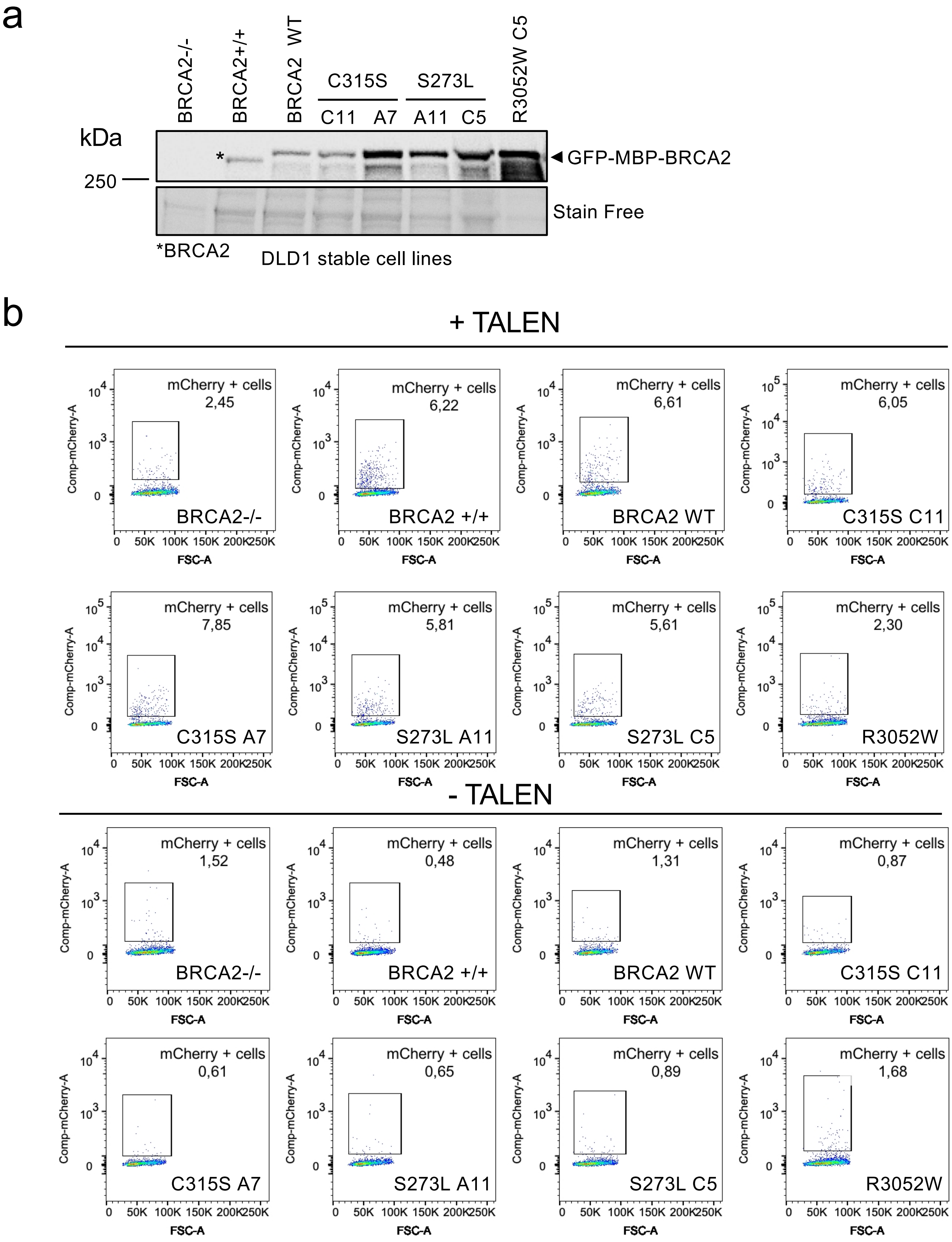
a
BRCA2-/-
BRCA2+/+
BRCA2 WT
C315S
C11
A7
S273L
A11
C5
R3052W C5
kDa
250
*
GFP-MBP-BRCA2
Stain Free
*BRCA2
DLD1 stable cell lines
b
+ TALEN
mCherry + cells 2,45 BRCA2-/-
mCherry + cells 6,22 BRCA2 +/+
mCherry + cells 6,61 BRCA2 WT
mCherry + cells 6,05 C315S C11
mCherry + cells 7,85 C315S A7
mCherry + cells 5,81 S273L A11
mCherry + cells 5,61 S273L C5
mCherry + cells 2,30 R3052W
- TALEN
mCherry + cells 1,52 BRCA2-/-
mCherry + cells 0,48 BRCA2 +/+
mCherry + cells 1,31 BRCA2 WT
mCherry + cells 0,87 C315S C11
mCherry + cells 0,61 C315S A7
mCherry + cells 0,65 S273L A11
mCherry + cells 0,89 S273L C5
mCherry + cells 1,68 R3052W
Comp-mCherry-A
FSC-A
0 50K 100K 150K 200K 250K

Supplementary Figure 2. Relative to Figure 1d. **(A)** BRCA2 protein levels in total cell extracts from DLD1 cells stably expressing EGFP-MBP-BRCA2 WT (BRCA2 WT) or the variants C315S (clones C11 and A7), S273L (clones A11 and C5), and R3052W (clone C5) as detected by western blot using anti-BRCA2 (OP95) antibody. (**B**) Example of the flow cytometry gating and analysis of mCherry positive cells as a readout from the HR assay at eight days post-transfection in DLD1 parental cells (BRCA2+/+), BRCA2 deficient cells (BRCA2-/-), or BRCA2-/- cells expressing BRCA2 WT, C315S A11, C315S A7, S273L A11, S273L C5, R3050W C5 as indicated. Cells only transfected with the donor plasmid without the TALEN plasmids are used as controls.

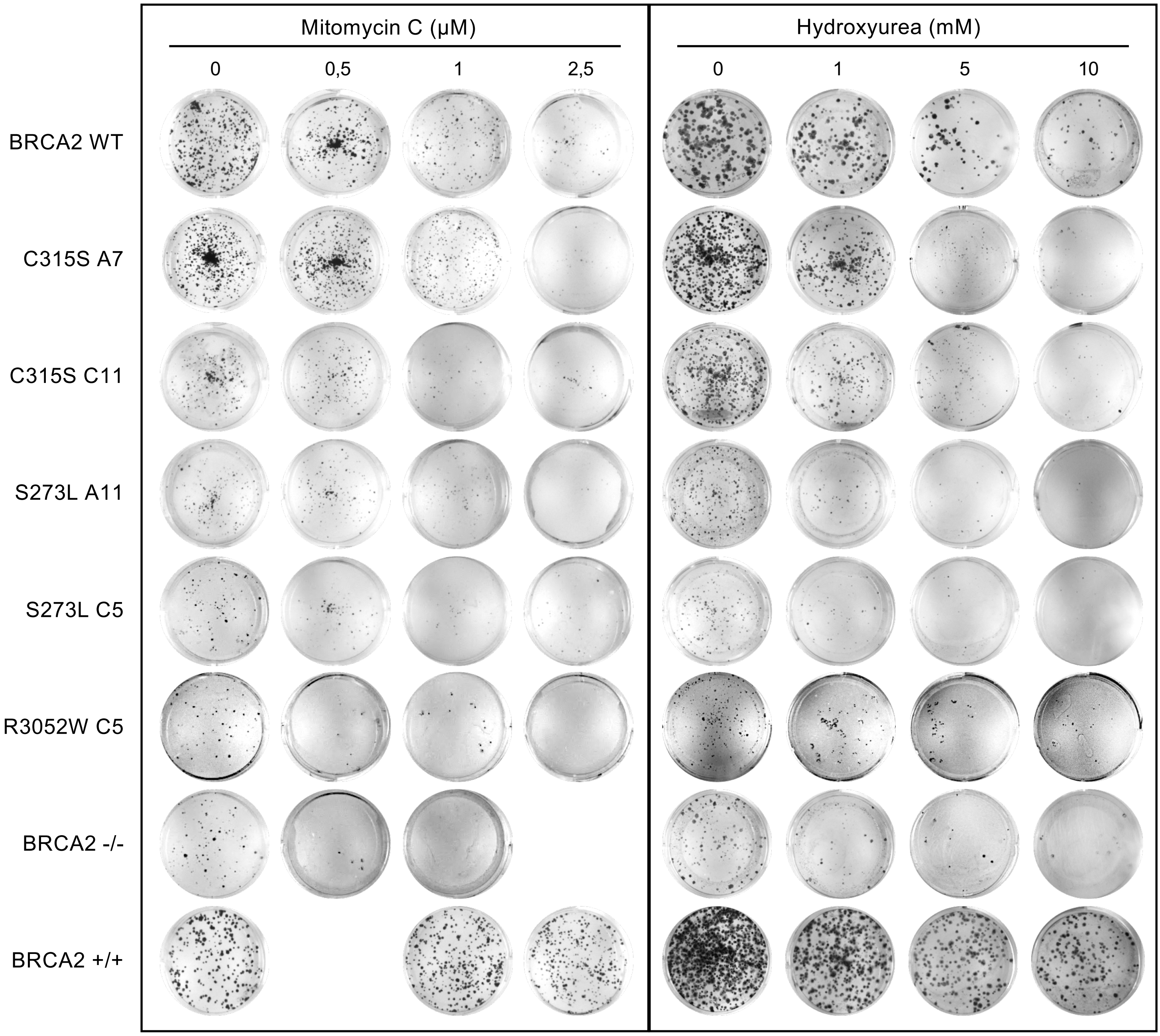
Mitomycin C (μM)
0
0,5
1
2,5
Hydroxyurea (mM)
0
1
5
10
BRCA2 WT
C315S A7
C315S C11
S273L A11
S273L C5
R3052W C5
BRCA2 -/-
BRCA2 +/+

Supplementary Figure 3. Relative to Figure 1e, f. **(A) (left)** Representative images of the clonogenic survival assay showing the plating efficiency in cells left untreated versus cells treated with 0.5, 1, or 2.5µM MMC, as indicated. The seeding number for the untreated cells was 500 cells per well for all clones except for BRCA2-/- and R3052W where it was 1000 cells. **(right)** Clonogenic survival assay showing the plating efficiency in cells left untreated versus cells treated with 1, 5, or 10mM HU, as indicated. The seeding number for the untreated cells was 500 cells per well for all clones except R3052W where it was1000. These experiments were performed in triplicates but only one well for each condition is shown for clarity.

a

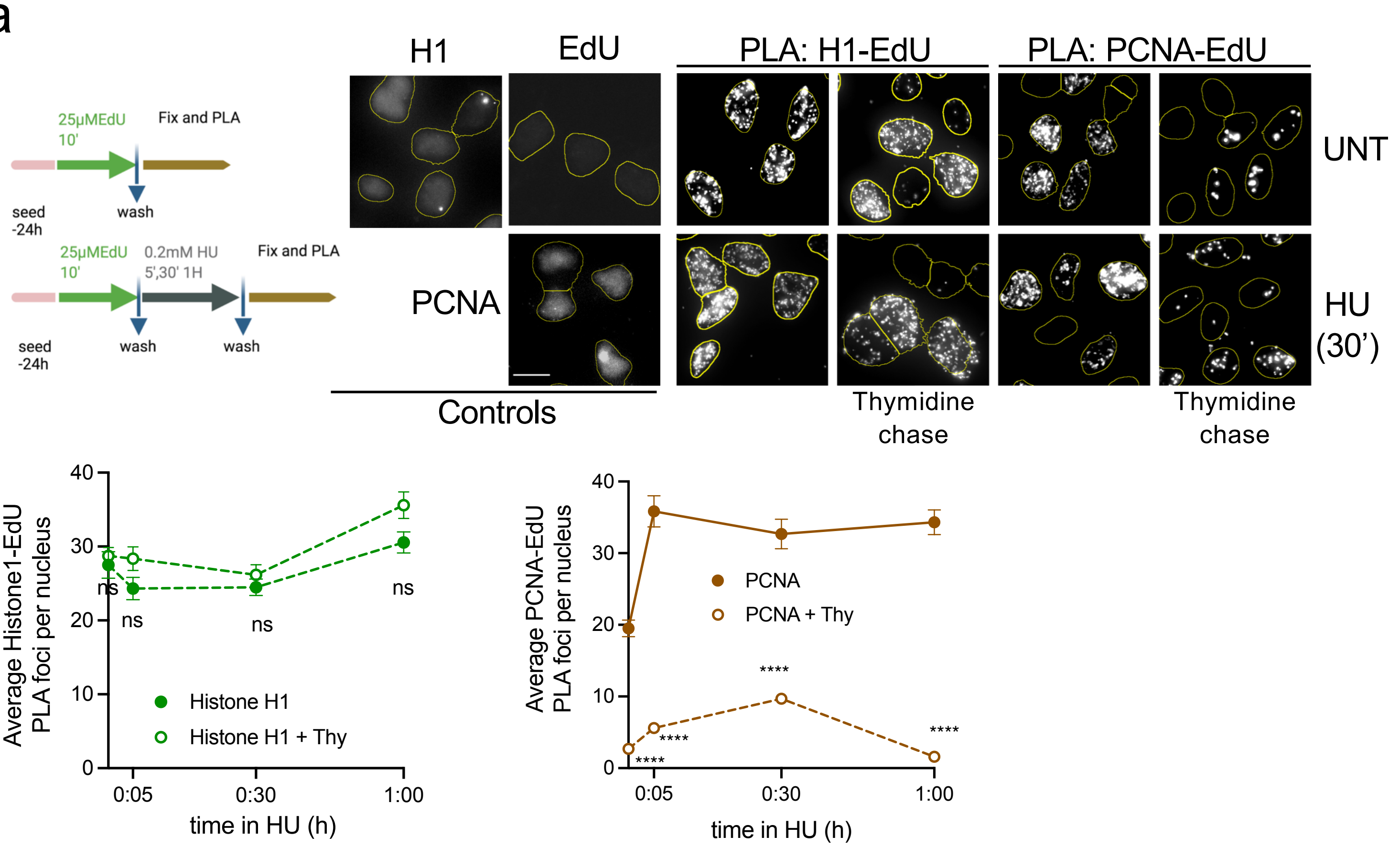
25μMEdU
10'
Fix and PLA
seed
-24h
wash
25μMEdU
10'
0.2mM HU
5,30' 1H
Fix and PLA
seed
-24h
wash
wash
H1
EdU
PLA: H1-EdU
PLA: PCNA-EdU
UNT
PCNA
HU
(30')
Controls
Thymidine
chase
Thymidine
chase
Average Histone1-EdU
PLA foci per nucleus
Histone H1
Histone H1 + Thy
ns
ns
ns
ns
time in HU (h)
0:05
0:30
1:00
Average PCNA-EdU
PLA foci per nucleus
PCNA
PCNA + Thy
****
****
****
****
time in HU (h)
0:05
0:30
1:00

b

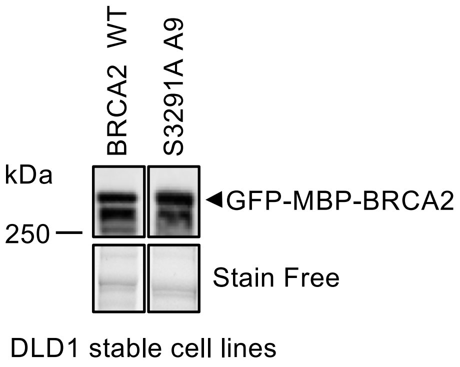
BRCA2 WT
S3291A A9
kDa
250
GFP-MBP-BRCA2
Stain Free
DLD1 stable cell lines

c

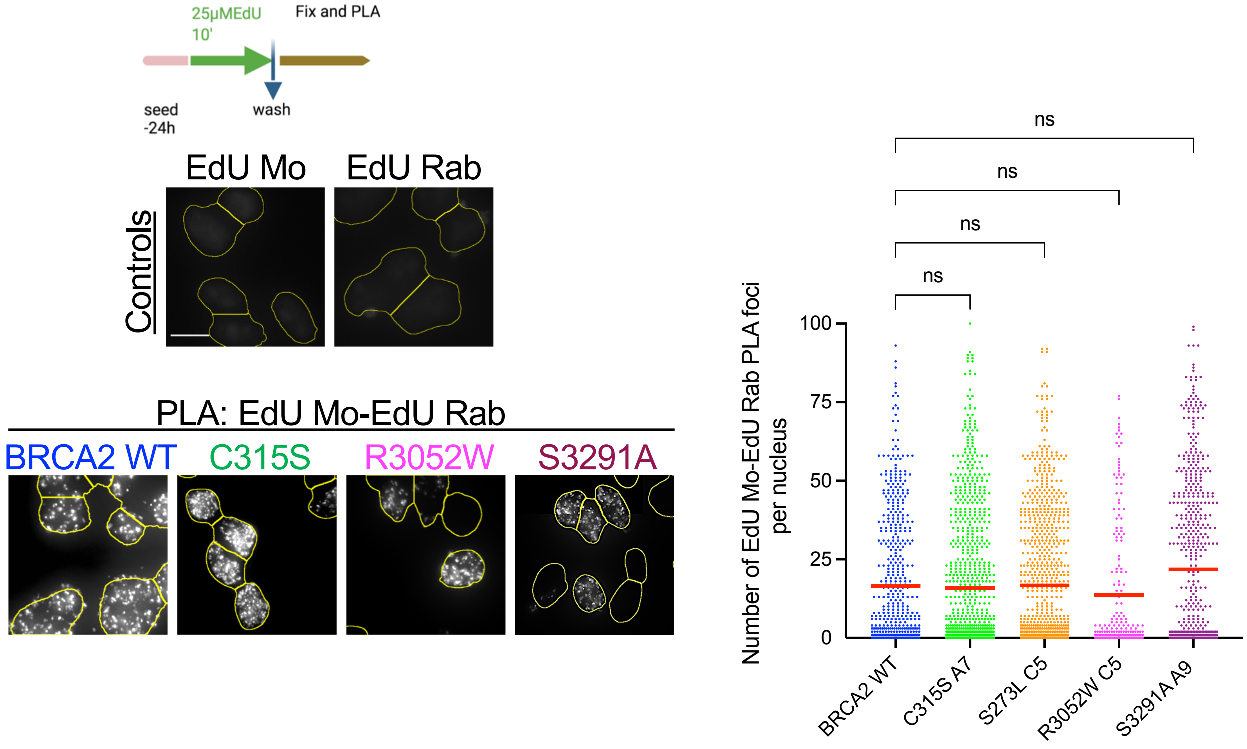
25μMEdU
10'
Fix and PLA
seed
-24h
wash
EdU Mo
EdU Rab
Controls
PLA: EdU Mo-EdU Rab
BRCA2 WT
C315S
R3052W
S3291A
Number of EdU Mo-EdU Rab PLA foci
per nucleus
ns
ns
ns
ns
BRCA2 WT
C315S A7
S273L C5
R3052W C5
S3291A A9

Supplementary Figure 4. Relative to Figure 2 and 3. **(a) (top left)** Scheme of the assay. **(top right)** Representative images of in situ PLA on nascent DNA between biotinylated EdU detected with anti-biotin antibody, PCNA, and histone H1 antibodies in DLD1 BRCA2 WT after 4h thymidine chase in untreated or after 30 min 0.2 mM HU treatment. The scale bar indicates 10 µm. **(bottom left)** Quantification of Histone H1 recruitment measured as the number of PLA foci between biotinylated EdU and Histone H1 observed per nucleus after 4h thymidine chase in BRCA2 WT cells. Data are represented as the median of two independent experiments with 200-300 cells analyzed in each experimental data set. Statistical difference was determined by the Kruskal-Wallis test followed by Dunn's multiple comparison test (ns, not significant). **(bottom right)** Quantification of the PCNA recruitment measured as the number of PLA foci between biotinylated EdU detected with anti-biotin antibody and PCNA observed per nucleus after 4h thymidine chase in BRCA2 WT cells. Data are represented as the median of two independent experiments with 200-300 cells analyzed in each experimental data set. Statistical difference was determined by the Kruskal-Wallis test followed by Dunn's multiple comparison test (****$p < 0.0001$). **(b)** Western blot showing the protein levels of the selected stable clone S3291A (A9) in comparison to the wild-type BRCA2 (WT) clone. The loading control is a cropped picture of the stain-free gel taken before transfer. **(c) (left)** Scheme of the assay and representative images of in situ PLA on nascent DNA between EdU Biotin conjugated mouse and EdU Biotin conjugated rabbit antibodies in DLD1 BRCA2 WT, the variants C315S (A7), S273L (C5), R3052W, and S3291A mutant in untreated conditions. For all the experiments we carried out two single-antibody control (EdU/Biotin mouse and rabbit) to assess the specificity of the PLA signal. **(right)** Quantification of the number of PLA foci observed per nucleus in untreated conditions. Data represent independent

experiments (BRCA2 WT n=3, C315S n=3, S273L n=1, S3291A n=2, R3052W n=2) with 200-300 cells analyzed in each experimental data set. Each PLA focus is one dot in the graph. Statistical difference was determined by the Kruskal-Wallis test followed by Dunn's multiple comparison test (ns, not significant) Scheme of the PLA assay created with BioRender.com.

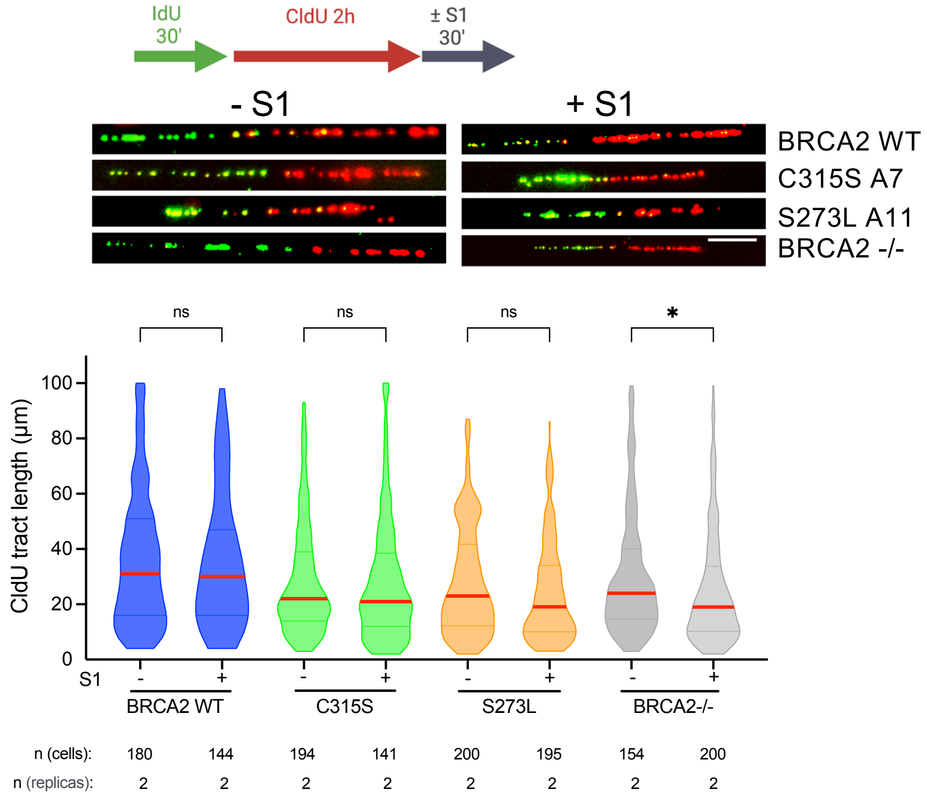

IdU
30'
CldU 2h
± S1
30'
- S1
+ S1
BRCA2 WT
C315S A7
S273L A11
BRCA2 -/-
ns
ns
ns
*
CldU tract length (µm)
100
80
60
40
20
0
S1
-
+
BRCA2 WT
C315S
S273L
BRCA2-/-
n (cells): 180 144 194 141 200 195 154 200
n (replicas): 2 2 2 2 2 2 2 2

Supplementary Figure 5. Relative to Figure 5**. (a) (Top)** Labelling scheme of thymidine analogs (IdU and CldU) and representative images of the replication tracks labelled as indicated from BRCA2-deficient cells (BRCA2-/-) alone or stably expressing either BRCA2-WT, BRCA2-C315S A7 or BRCA2-S273L A11. The scale bar indicates 10 µm. **(b)** Quantification of CldU track length in cells from (**Top**). Data represent the median + 25% and 75% quartiles of two independent experiments with the following number of fibers (CldU) analysed: (BRCA2 WT -S1 sample size = 180, BRCA2WT+S1 sample size = 144, C315S -S1 sample size = 194, C315S +S1 sample size = 141, S273L -S1 sample size = 200, S273L +S1 sample size = 195, BRCA2-/- -S1 sample size = 154, BRCA2-/- +S1 sample size = 200). Statistical difference was determined by the Kruskal-Wallis test followed by Dunn's multiple comparison test. ns, not significant, $^{*}p<0.05$ ($p=0.0271$).

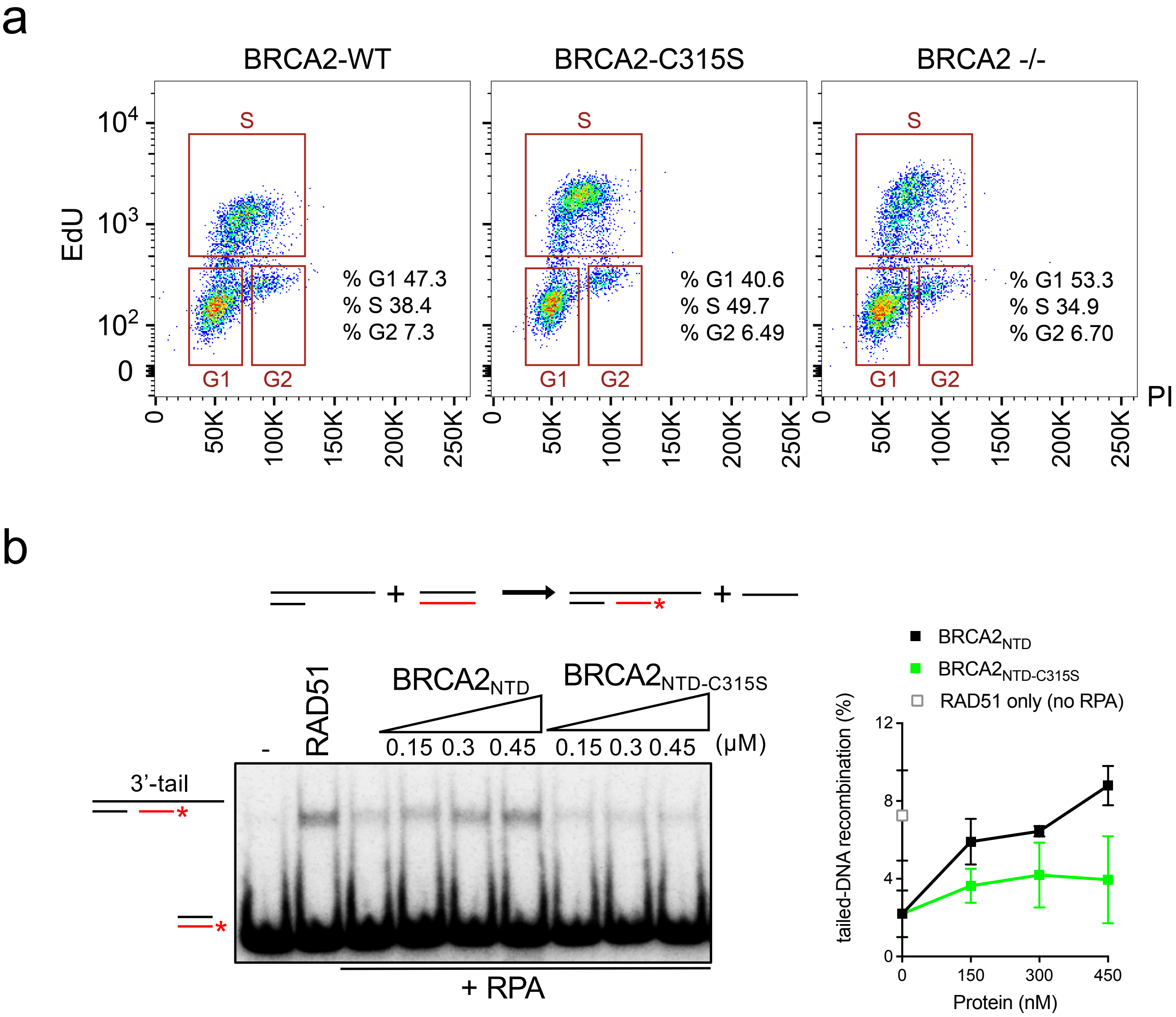
a
BRCA2-WT
BRCA2-C315S
BRCA2 -/-
EdU
PI
S
G1
G2
% G1 47.3
% S 38.4
% G2 7.3
% G1 40.6
% S 49.7
% G2 6.49
% G1 53.3
% S 34.9
% G2 6.70
b
3'-tail
-
RAD51
BRCA2NTD
BRCA2NTD-C315S
0.15 0.3 0.45
0.15 0.3 0.45
(μM)
+ RPA
BRCA2NTD
BRCA2NTD-C315S
RAD51 only (no RPA)
tailed-DNA recombination (%)
Protein (nM)

Supplementary Figure 6. Relative to Figure 6. (**a**). Representative flow cytometry plots of cells stained with anti-EdU antibody and Propidium Iodide/PI (DNA). (**b)** DNA strand exchange reaction using a resected mimicking substrate (Suppl. Table 1) in the presence or absence of RPA, RAD51, and increasing concentrations of $BRCA2_{NTD\text{-}WT}$ or $BRCA2_{NTD\text{-}C315S}$, as indicated. **(Right)** Quantification of the reaction on the left. Data represent the mean from three independent experiments. Error bars, SD.

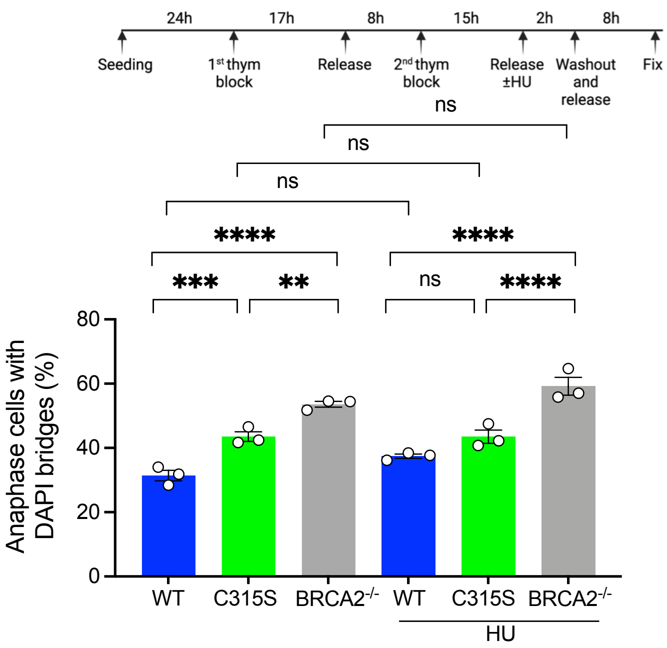
24h
17h
8h
15h
2h
8h
Seeding
1st thym block
Release
2nd thym block
Release ±HU
Washout and release
Fix
ns
ns
ns
****
****
***
**
ns
****
Anaphase cells with DAPI bridges (%)
80
60
40
20
0
WT
C315S
BRCA2-/-
WT
C315S
BRCA2-/-
HU

Supplementary Figure 7. Relative to Figure 7. (**Top**). Scheme of the synchronization procedure used to analyse anaphase bridges. **(Bottom)** Quantification of cells with aberrant chromosomes segregation in BRCA2−/− cells and in the BRCA2−/− clones stably expressing BRCA2 WT, BRCA2-C315S (A7), as indicated in untreated conditions or after 2h treatment with HU (0.5mM). Data represent the mean and SEM of three independent experiments: The total number of anaphases analyzed in the 3 biologically independent experiments was 476 (BRCA2 WT), 456 (C315S), 458 ($BRCA2^{-/-}$), 483 (WT+HU), 476 (C315S+HU) and 463 ($BRCA2^{-/-}$+HU). A two-way ANOVA test with Tukey's multiple comparisons test was used to calculate the statistical significance of the differences (normal vs cells with anaphase bridges, only cells with anaphase bridges are plotted in the graph).

Supplementary Table 1: Oligonucleotides used in this study

Primer/oligo	Purpose	Sequence (5'-3')
oAC403	3'tail and gapped DNA substrate in strand exchange assay, anneals with oAC423	CGGATATTTCTGATGAGTCGAAAAATTATCT TGATAAAGCAG
oAC423	3'tail and gapped DNA substrate in strand exchange assay, anneals with oAC403 and oAC490	CTGCTTTATCAAGATAATTTTTCGACTCATCA GAAATATCCGTTTCCTATATTTATTCCTATTA TGTTTTATTCATTTACTTATTCTTTATGTTCAT TTTTTATATCCTTTACTTTATTTTCTCTGT TTATTCATTTACTTATTTTGTATTATCCTTATC TTATTTA
oAC490	gapped DNA substrate in strand exchange assay, anneals with oAC423	TAAATAAGATAAGGATAATACAAAATAAGTA AATGAATAAAC
oAC1076	dsDNA for strand exchange assay, anneals with oAC1077	ATAAAAAATGAACATAAAGAATAAGTAAATG AATAAAACAT
oAC1077	dsDNA for strand exchange assay, anneals with oAC1076	ATGTTTTATTCATTTACTTATTCTTTATGTTCA TTTTTTAT
oAC379	dT40 for EMSA and biotin pull-down	TTTTTTTTTTTTTTTTTTTTTTTTTTTTTTTTTTT TTTTT
oAC405	40mer for EMSA, anneals with oAC406	TAATACAAAATAAGTAAATGAATAAACAGAG AAAATAAA G
oAC406	40mer for EMSA, anneals with oAC405	CTTTATTTTCTCTGTTTATTCATTTACTTATTT TGTATTA
oAC596	Primer #1 to produce 191bp PCR product used in EMSA	TTCCAGGGGCCCGGCGGCCGCGATCCAAA AAAGAAGAGA AAGG
oAC597	Primer #2 to produce 191bp PCR product used in EMSA	GTGTTTTCACTTTGCTCTTTTTCATCAAAAAG G
