# Supplementary material for: Replication gap suppression depends on the double-strand DNA binding activity of BRCA2
Source: Nat Commun. 2023 Jan 27;14:446. doi: 10.1038/s41467-023-36149-0 (PMC9883520; doi:10.1038/s41467-023-36149-0)
Supplement: Supplementary file 3 — Reporting Summary [file 41467_2023_36149_MOESM3_ESM.pdf]

nature portfolio

Corresponding author(s): Aura Carreira

Last updated by author(s): 20-12-2022

Reporting Summary

Nature Portfolio wishes to improve the reproducibility of the work that we publish. This form provides structure for consistency and transparency in reporting. For further information on Nature Portfolio policies, see our Editorial Policies and the Editorial Policy Checklist.

Statistics

For all statistical analyses, confirm that the following items are present in the figure legend, table legend, main text, or Methods section.

n/a	Confirmed	
☐	☒	The exact sample size (*n*) for each experimental group/condition, given as a discrete number and unit of measurement
☐	☒	A statement on whether measurements were taken from distinct samples or whether the same sample was measured repeatedly
☐	☒	The statistical test(s) used AND whether they are one- or two-sided<br>*Only common tests should be described solely by name; describe more complex techniques in the Methods section.*
☒	☐	A description of all covariates tested
☐	☒	A description of any assumptions or corrections, such as tests of normality and adjustment for multiple comparisons
☐	☒	A full description of the statistical parameters including central tendency (e.g. means) or other basic estimates (e.g. regression coefficient) AND variation (e.g. standard deviation) or associated estimates of uncertainty (e.g. confidence intervals)
☐	☒	For null hypothesis testing, the test statistic (e.g. *F*, *t*, *r*) with confidence intervals, effect sizes, degrees of freedom and *P* value noted<br>*Give P values as exact values whenever suitable.*
☒	☐	For Bayesian analysis, information on the choice of priors and Markov chain Monte Carlo settings
☒	☐	For hierarchical and complex designs, identification of the appropriate level for tests and full reporting of outcomes
☒	☐	Estimates of effect sizes (e.g. Cohen's *d*, Pearson's *r*), indicating how they were calculated

Our web collection on statistics for biologists contains articles on many of the points above.

Software and code

Policy information about availability of computer code

Data collection	Metamorph 7.10.4.407 software (Molecular Devices) was used to acquire in situ Proximity Ligation Assay imaging data and DNA combing imaging; Replication tracks in DNA fiber assay were acquired using Zeiss Zen 3.1 software. FACSDiva software 6.0 was used for the acquisition of cell cycle data and mCherry positive cells
Data analysis	Flow cytometry data was analysed using FlowJo Software version 10.5 software (Tree Star Inc). Image data was analysed using ImageJ (1.52k NIH, https://imagej.nih.gov). Radiactive label of the in vitro recombination and EMSA experiments were analysed with ImageQuant TL Software v8.2.0(GE Healthcare) software. For statistical analysis and graphical data presentation we used Prism (version Mac OS X 9.4.0 (453)) GraphPad Software.

For manuscripts utilizing custom algorithms or software that are central to the research but not yet described in published literature, software must be made available to editors and reviewers. We strongly encourage code deposition in a community repository (e.g. GitHub). See the Nature Portfolio guidelines for submitting code & software for further information.

Data

Policy information about availability of data

All manuscripts must include a data availability statement. This statement should provide the following information, where applicable:

- Accession codes, unique identifiers, or web links for publicly available datasets
- A description of any restrictions on data availability
- For clinical datasets or third party data, please ensure that the statement adheres to our policy

The data that support this study are available in the Source File as supplementary information

Human research participants

Policy information about studies involving human research participants and Sex and Gender in Research.

Reporting on sex and gender	n/a
Population characteristics	n/a
Recruitment	n/a
Ethics oversight	n/a

Note that full information on the approval of the study protocol must also be provided in the manuscript.

Field-specific reporting

Please select the one below that is the best fit for your research. If you are not sure, read the appropriate sections before making your selection.

☒ Life sciences ☐ Behavioural & social sciences ☐ Ecological, evolutionary & environmental sciences

For a reference copy of the document with all sections, see nature.com/documents/nr-reporting-summary-flat.pdf

Life sciences study design

All studies must disclose on these points even when the disclosure is negative.

Sample size	No sample size calculation was performed. We chose the sample size to obtain a representative number of cells from several independent experiments to get enough statistical power.
Data exclusions	No data has been excluded
Replication	All the experiments were reliably reproduced, the number of independent experiments are specified in the figure legends for all experiments.
Randomization	For all cell based experiments the control and the treated cells are coming from the same populations and treated at the same time; the comparison of different cell lines/clones was performed using similar passage of the cells.
Blinding	All the immunofluorescence experiments were performed blinded. All other experiments were blinded during data collection.

Reporting for specific materials, systems and methods

We require information from authors about some types of materials, experimental systems and methods used in many studies. Here, indicate whether each material, system or method listed is relevant to your study. If you are not sure if a list item applies to your research, read the appropriate section before selecting a response.

Materials & experimental systems

n/a	Involved in the study
☐	☒ Antibodies
☐	☒ Eukaryotic cell lines
☒	☐ Palaeontology and archaeology
☒	☐ Animals and other organisms
☒	☐ Clinical data
☒	☐ Dual use research of concern

Methods

n/a	Involved in the study
☒	☐ ChIP-seq
☐	☒ Flow cytometry
☒	☐ MRI-based neuroimaging

Antibodies

Antibodies used

Primary antibodies used for PLA were as follows: BRCA2 (1:500 OP95 EMD Millipore), Biotin (1:3000 Bethyl laboratories), Biotin (1:3000 Jackson ImmunoResearch), RAD51 (1:500 Novus Biologicals), PCNA (1:500 Santa Cruz Biotechnology) and Histone H1 (1:500 Santa Cruz Biotechnology). Antibodies used for Western blotting: Antibodies used for western blotting
Mouse anti-MBP (1:5000, R29, Cat. #MA5-14122, Thermo Fisher Scientific), mouse anti-BRCA2 (1:1000, OP95, EMD Millipore), mouse anti-CHK1 (1:1000, Cat. #2360, Cell Signaling Technology), rabbit anti-pCHK1-S345 (1:500, Cat #2348, Cell Signaling Technology), Horseradish peroxidase (HRP) conjugated secondary antibodies used: mouse-IgGκ BP-HRP (IB: 1:5000, Cat. #sc-516102, Santa Cruz), HRP Goat anti-mouse IgG (1:10000, Cat #115-035-003, Jackson Immuno), HRP Goat anti-rabbit IgG (1:10000, Cat #111-035-003, Jackson Immuno). DNA fiber assay antibodies: IdU replication tracts were revealed with a mouse anti-BrdU/IdU antibody from BD Biosciences (347580; 1:100) and CldU tracts with a rat anti-BrdU/CldU antibody from Eurobio (ABC117-7513; 1:100). The following secondary antibodies were used: Alexa fluor 488 anti-mouse antibody (Life A21241; 1:100) and Cy3 anti-rat antibody (Jackson ImmunoResearch 712-166-153; 1:100). Antibodies for DNA combing: primary anti-IdU and anti-CldU antibodies (1:25 anti-mouse Becton Dickinson 347580 for IdU and 1:50 anti-rat Abcam ab6326 for CIdU); secondary antibodies: 1:50 Alexa donkey anti-mouse 488 (Life Technologies ref. 21202), 1:50 Alexa goat anti-rat 555 (Life Technologies ref. A21434).

Validation

Validations are based on the datasheets from the manufacturers (RRIDs of the antibodies are provided), as indicated below:
Proximity Ligation Assay:
Biotin (1:3000 Bethyl laboratories), BETA150-109A
• Species reactivity: N/A
• Applications: ELISA, ICC, IHC, WB
• Isotype : IgG

Biotin (1:3000 Jackson ImmunoResearch), AB_2339006
• Species reactivity: N/A
• Applications: N/A
• Isotype : IgG

RAD51 (1:500 Novus Biologicals), NB100-148
• Species reactivity: Human, Mouse, Rat, C. elegans, Chicken
• Applications: WB, ChIP, ICC/IF, IHC, IHC-Fr, IHC-P, In vitro, IP, PLA, KD
• Isotype : IgG2b

PCNA (1:500 Santa Cruz Biotechnology), sc-56
• Species reactivity: mouse, rat, human, insect and S. pombe
• Applications: WB, IP, IF, IHC(P) et FCM
• Isotype : IgG2a

Histone H1 (1:500 Santa Cruz Biotechnology), sc-8030
• Species reactivity: "broad species"
• Applications: WB, IP, IF, IHC(P), FCM et ELISA
• Isotype : Mouse IgG2a

Western-blot:
Mouse anti-MBP (1:5000, R29, Cat. #MA5-14122, Thermo Fisher Scientific)
• Species reactivity: N/A
• Applications: IF and WB
• Source/Isotype: Mouse IgG1

Mouse anti-BRCA2 (1:1000, OP95, EMD Millipore)
• Species reactivity: Human
• Applications: WB, IP
• Source/Isotype: Mouse IgG2b

Mouse anti-CHK1 (1:1000, Cat. #2360, Cell Signaling Technology)
• Species reactivity: Human, Mouse, Rat, Monkey
• Applications: WB, IP, IHC, ChIP, C&R, C&T, DB, eCLIP, IF, Flow Cytometry
• Source/Isotype: Mouse IgG1

Rabbit anti-pCHK1-S345 (1:500, Cat #2348, Cell Signaling Technology)
• Species reactivity: Human, Mouse, Rat, Monkey
• Applications: WB, IP, IHC, ChIP, C&R, C&T, DB, eCLIP, IF, Flow Cytometry
• Source/Isotype: Rabbit IgG

DNA fiber :
Mouse Anti-BrdU for IdU (1:100, #347580, BD Biosciences)
• Species reactivity: N/A
• Applications: Flow Cytometry
• Source/Isotype: Mouse IgG1

Mouse Anti-BrdU for CldU (1:100, ABC117-7513, Eurobio)
• Species reactivity: N/A
• Applications: Immunohistochemistry
• Source: Mouse

DNA combing:
Primary anti-IdU (anti-mouse Becton Dickinson 347580) as for DNA fiber

Primary anti-CldU (anti-rat Abcam ab6326)

Species reactivity: reacts with species independent
Application: ICC/IF, IHC-P, Flow Cyt
Source/Isotype: rat IgG2a

Eukaryotic cell lines

Policy information about cell lines and Sex and Gender in Research

Cell line source(s)	The BRCA2 deficient colorectal adenocarcinoma cell line DLD1 BRCA2-/- (Hucl, T. et al 2008) (HD 105-007) and the parental cell line DLD1 BRCA2+/+ (HD-PAR-008) was purchased from Horizon Discovery (Cambridge, England). The human cell line HEK293T (kind gift from Dr. Mounira Amor-Gueret) (Institut Curie, Orsay, FR) was originally purchased from ATCC
Authentication	The authentication of all cells was performed by genotyping using GenePrint kit (Promega).
Mycoplasma contamination	All cell lines used in this study have been regularly tested for mycoplasma contamination (MycoAlert, Lonza) and are mycoplasma-free
Commonly misidentified lines (See ICLAC register)	The only commonly misidentified cell-line used in this study is the HEK293T that we only used for protein purifications and the cells have been genotyped.

Flow Cytometry

Plots

Confirm that:

☒ The axis labels state the marker and fluorochrome used (e.g. CD4-FITC).

☒ The axis scales are clearly visible. Include numbers along axes only for bottom left plot of group (a 'group' is an analysis of identical markers).

☒ All plots are contour plots with outliers or pseudocolor plots.

☒ A numerical value for number of cells or percentage (with statistics) is provided.

Methodology

Sample preparation	To label replicated DNA, cells were incubated with 10µM EdU for 2h. Samples were collected by trypsinization and incorporated EdU was detected using the Click-iT EdU Alexa Fluor 647 Flow Cytometry Assay Kit (Molecular Probes-Thermofisher Scientific) according to manufacturer's instructions. Cells were re-suspended in PBS containing 20µgml−1 propidium iodide (Sigma) and 10µgml−1 RNase A (Sigma) before samples were processed using flow cytometry For Homologous Recombination gene targeting assay DLD1 BRCA2−/− cells stably expressing full-length GFP-MBP-BRCA2 and the variants (C315S, S273L, and R3052W) were transfected using AMAXA technology (Lonza) nucleofector kit V (Cat. #VCA-1003) with 3µg of the promoter-less donor plasmid (AAVS1-2A-mCherry) with or without 1µg of each AAVS1-TALEN encoding plasmids (TALEN-AAVS1-5′ and TALEN-AAVS1-3′, a kind gift from Dr. Carine Giovannangeli). The day after transfection the media was changed and 48h post-transfection the cells were trypsinized and reseeded on a 10-cm culture dish and cultured for additional 8 days.

Instrument	Replicated DNA (BD FACSCalibur, BD Biosciences), HR gene targeting : The percentage of mCherry positive cells was analyzed on a BD FACSAria III (BD Bioscience) using the FACSDiva software
Software	Data were analysed using FlowJo 10.5 software (Tree Star Inc.).
Cell population abundance	10,000 events of FSC singlets (gated in FSC-W vs FSC-A plot) were collected for each experiment, the whole FSC singlet cell population were analysed for EdU. For HR gene targeting assay: Viable and single cells were gated using forward scatter (FSC-A) and side scatter (SSC-S). To separate single cells from the doublets, singlets were selected using FSC-W(y-axis) plotted against FSC-A(x-axis), mCherry positive cells were detected by plotting mCherry-A(y-axis) against FSC-A(x-axis).
Gating strategy	Viable and single cells were gated using forward scatter (FSC-A) and side scatter (SSC-S). To separate single cells from the doublets, singlets were selected using FSC-W(y-axis) plotted against FSC-A(x-axis), mCherry positive cells were detected by plotting mCherry-A(y-axis) against FSC-A(x-axis). The gating for mCherry was based on non-transfected cells of the same cell line. EdU positive cells were detected by plotting EdU (y-axis) against Propidium Iodide (x-axis). EdU+ was based on non-stained cells.

☒ Tick this box to confirm that a figure exemplifying the gating strategy is provided in the Supplementary Information.
